# Role of FXR in Renal Physiology and Kidney Diseases

**DOI:** 10.3390/ijms24032408

**Published:** 2023-01-26

**Authors:** Yanlin Guo, Guixiang Xie, Xiaoyan Zhang

**Affiliations:** Health Science Center, East China Normal University, Shanghai 200241, China

**Keywords:** Farnesoid X receptor, renal physiology, acute kidney disease, chronic kidney disease

## Abstract

Farnesoid X receptor, also known as the bile acid receptor, belongs to the nuclear receptor (NR) superfamily of ligand-regulated transcription factors, which performs its functions by regulating the transcription of target genes. FXR is highly expressed in the liver, small intestine, kidney and adrenal gland, maintaining homeostasis of bile acid, glucose and lipids by regulating a diverse array of target genes. It also participates in several pathophysiological processes, such as inflammation, immune responses and fibrosis. The kidney is a key organ that manages water and solute homeostasis for the whole body, and kidney injury or dysfunction is associated with high morbidity and mortality. In the kidney, FXR plays an important role in renal water reabsorption and is thought to perform protective functions in acute kidney disease and chronic kidney disease, especially diabetic kidney disease. In this review, we summarize the recent advances in the understanding of the physiological and pathophysiological function of FXR in the kidney.

## 1. Introduction

Nuclear receptors are a superfamily of ligand-dependent transcription factors, with typical domain structures and conserved sequences. There are 48 members of the human nuclear receptor superfamily which perform crucial roles in a variety of processes of physiology and pathophysiology, such as homeostasis, reproduction, development, metabolism, obesity, diabetes, hypertension and cancer. Therefore, they always attract attention from basic scientists, clinicians, and the pharmaceutical industry, and account for 3% of all human drug targets so far [1].

Nuclear receptor farnesoid X receptor (FXR) was first isolated from a rat liver cDNA library and named after its weak activation via supraphysiological farnesol, an intermediate in the mevalonate biosynthetic pathway, in 1995 [2,3]. Subsequently, bile acids were identified as endogenous FXR ligands and were given another name: bile acid receptors. As a bile acid sensor, FXR is a key modulator of enterohepatic circulation of bile acids, controlling transcription of key regulatory genes in bile acid synthesis, biliary bile acid secretion and trans-intestinal bile acid transport to the liver via portal blood circulation. FXR also serves as a metabolic regulator of glucose, lipid and energy metabolism. Because of its master functions in bile acid and lipid metabolism, FXR is considered a promising drug target for the therapy of bile-acid-related liver diseases. In 2016, obeticholic acid (OCA), a highly selective agonist of FXR, was approved by the U.S. Food and Drug Administration for treatment of primary biliary cholangitis (PBC), a chronic cholestatic liver disease. Currently, FXR agonists are tested in clinical trials for treatment of metabolic and gastrointestinal diseases, including type 2 diabetes (T2DM), metabolic syndrome and Non-Alcoholic Steatohepatitis (NASH).

The kidney is a key organ, managing water and solute homeostasis for the whole body, and kidney injury or dysfunction is associated with high morbidity and mortality. FXR exhibits high expression in the kidney, but little is known about its role in the kidney. Recently, we and other groups reported that FXR participates in renal water reabsorption and is involved in kidney diseases, including acute kidney injury and chronic kidney disease. This review summarizes the structure and expression of FXR, with a particular focus on the recent advancement of FXR in regulation of renal physiology and kidney diseases. 

## 2. The Structure and Expression of FXR

Two FXR protein families have been identified in animals. Derived from the nuclear receptor subfamily 1, group H, member *(NR1H) 4* and *NR1H5* genes, they are named FXRα and FXRβ, respectively. From fish to humans, FXRα is evolutionarily conserved among species. Due to the activation of two different promoters and the use of alternative splicing, the FXRα gene encodes four isoforms (FXRα1-α4) [4,5]. A recent study identified four novel splice variants (FXRα5–8) in human hepatocytes, which resulted from previously undetected exon skipping events. The possible roles of these novel isoforms in human liver require further investigation [6]. However, the FXRβ coding gene is a pseudogene in humans and primates [7]. FXR is highly expressed in the liver, intestine, kidney, and adrenal gland, with low levels of expression in adipose tissue and heart. The expression of FXRα1-α4 in different tissues remains unclear. Using Southern blot analysis, Zhang et al. found that mouse FXRα1-α4 had the highest expression in liver, and the expression levels of each isoform are similar. In addition, FXRα1 and FXRα2 are moderately expressed in the ileum and adrenal gland; FXRα3 and FXRα4 are highly expressed in the ileum and moderately expressed in the kidney [8]. More efforts are needed to analyze the tissue distribution of different FXR isoforms in the future to help elucidate their functions (Table 1).

FXR has a typical domain structure of a nuclear receptor (NR), including an N-terminal domain (NTD), DNA-binding domain (DBD), hinge region and ligand-binding domain (LBD). The N-terminal domain (NTD) contains a ligand-independent transcriptional activation domain (AF1) which can interact with other coregulator proteins such as peroxisome proliferator-activated receptor gamma (PPARγ) coactivator 1-alpha (PGC1α), E1A-binding protein p300 (P300) and nuclear receptor co-repressor 2 (NCOR2/SMRT) [9], and many post-translational modifications (PTM) including phosphorylation, SUMOylation, GlcNAcylation, acetylation and methylation can occur in this region [10]. PGC-1α functions as a transcriptional coactivator of FXR that directly interacts with FXR and enhances its transactivation activity [11]. Phosphorylation of FXR by protein kinase C promotes its transcriptional activity [12]. FXR O-GlcNAcylation at S62 results in increased FXR activity [13]. Some coactivators have intrinsic enzymatic activity and can modify NRs themselves. Acetylation of FXR by P300 increases its stability but reduces FXR-RXRα heterodimerization, leading to a reduction in FXR target gene expression [14]. The zinc finger DNA-binding domain (DBD) is the most conserved domain, containing two zinc finger motifs that recognize specific DNA sequences [15]. The flexible hinge region is a linker between the DBD and the LBD, which is also a site for the regulation of PTMs such as the NTD [10]. Ligand-binding domain (LBD) is composed of 11 α-helices and four β-strands that fold into three parallel layers to form an alpha helical sandwich. This fold forms a hydrophobic C-terminal ligand binding pocket (LBP) and AF-2. LBD can bind to ligands and AF-2 can recruit co-activators [16,17,18]. When ligands are present, FXR binds to specific DNA sequences in the promoter of target genes in the form of a monomer or a heterodimer with retinoid X receptor (RXR), the common partner for NRs, to regulate gene transcription [19].

## 3. The Ligands of FXR

Bile acids are important endogenous agonists of FXR. Cholic acid (CA) and chenodeoxycholic acid (CDCA) are two primary bile acids synthesized in the liver. The secondary bile acids lithocholic acid (LCA) and deoxycholic acid (DCA) are generated from CA and CDCA. The potency of bile acids in activating FXR is ranked as: CDCA > DCA > LCA > CA [20]. OCA is a semi-synthetic derivative of CDCA, also known as 6-ethyl-CDCA and INT-747, which is the first FXR agonist to enter clinical research [21]. However, OCA has side effects such as dose-dependent pruritis, which can lead to treatment discontinuation in ~1–10% of patients. EDP-305 is another steroid FXR agonist studied for the treatment of NASH and PBC [22]. In order to reduce side effects and improve the therapeutic effect, non-steroidal FXR agonists have been gradually synthesized. These include GW4064 [23], Cilofexor [24], Tropifexor [25], Nidufexor [26] and others [27,28,29,30,31,32,33,34]. Therefore, FXR agonists are actively used to treat a variety of metabolic diseases in the clinics [35] (Table 2).

Some bile acids are considered FXR Antagonists. Tauro-β-muricholic acid (T-β-MCA), an endogenous FXR antagonist, was reported to inhibit activation of FXR [36]. It is reported that glycine-β-muricholic acid (Gly-MCA) inhibited FXR signaling exclusively in the intestine, resulting in a decrease in the serum and intestine ceramide level and an improvement in metabolic dysfunction in obese mice [37]. Ursodeoxycholic acid (UDCA) exerts FXR-antagonistic effects on bile acid and lipid metabolism in morbid obesity, although it is a commonly used therapeutic agent in cholestatic liver disease [38]. More recently, Brevini et al. found that UDCA reduces angiotensin-converting enzyme 2 (ACE2) expression by inhibiting FXR activity, resulting in reduced susceptibility to SARS-CoV-2 infection [39]. Therefore, FXR antagonists are also actively used to treat multiple diseases in the clinics (Table 3).

## 4. The General Function of FXR

### 4.1. FXR and Bile Acid Metabolism

FXR plays critical roles in bile acid homeostasis [46]. FXR inhibits bile acid synthesis: in the liver, FXR decreases the expression of cytochrome P450 (CYP)7A1 and CYP8A1, which are rate-limiting enzymes in bile acid biosynthesis of cholesterol [47,48]. In the intestinal enterocytes, FXR induces the expression of fibrotic growth factor 15 (FGF15; FGF19, the orthologue humans of FGF15), which travels through the portal vein to the liver and activates the FGF receptor 4 (FGFR4)/β-Klotho complex, thereby inhibiting transcription of CYP7A1 and CYP8A1 [49]. FXR reduces bile acid accumulation in hepatocytes and enterocytes: in liver, FXR can inhibit the expression of Na^+^-taurocholate cotransporting polypeptide (NTCP) and organic-anion-transporting polypeptides (OATP) at the sinusoidal membrane to reduce hepatocytes’ reabsorption of bile acids in the portal vein [50]. FXR promotes the excretion of bile acids into bile by activating transporters on the apical membrane surface, including multidrug-resistance-associated protein (MRP)2/3 [51], bile salt export pump (BSEP) [52] and multidrug resistance (MDR)2/3 [53]. FXR also promotes bile acid efflux into blood circulation by inducing expression of MRP4 and organic solute transporter (OST)α/OSTβ in the basolateral membrane [54]. In the intestine, FXR inhibits apical sodium-dependent bile acid transporter (ASBT) at the apical membrane surface [55], thereby reducing the reabsorption of bile acids in the intestinal epithelium, upregulating ileal bile acid-binding protein (IBABP) and promoting its movement from the apical membrane to the basolateral membrane [56]. Moreover, FXR promotes the transportation of bile acids to the portal vein and their subsequent return to the liver via upregulation of the expression of OSTα and OSTβ on the basolateral membrane surface of the enterocyte [57].

### 4.2. FXR and Glucose Metabolism

FXR plays diverse roles in glucose metabolism. The activation of FXR increases glycogen synthesis by inhibiting glycogen synthase kinase-3 beta (GSK3β) gene expression, which phosphorylates and subsequently inactivates glycogen synthase [58,59], reduces glycolysis by suppressing the transcriptional activity of ChREBP [60] and decreases the expression of multiple gluconeogenic genes, including phosphoenolpyruvate carboxykinase (PEPCK) and glucose 6-phosphatase (G-6-Pase) [58,61], resulting in a decrease in gluconeogenesis and serum glucose. However, several studies found that FXR antagonist exhibited a beneficial effect on glucose metabolism in T2DM mice, although the exact mechanism remains unclear [62]. Furthermore, FXR increases glucose-stimulated insulin secretion in islets [63] by inducing the expression of glucose-regulated transcription factor Krueppel-like factor 11 (KLF11) [64], adenylyl cyclase 8 (ADCY8) [65] and the transient receptor potential ankyrin 1 (TRPA1) channel [66].

### 4.3. FXR and Lipid Metabolism

**Figure 1:**
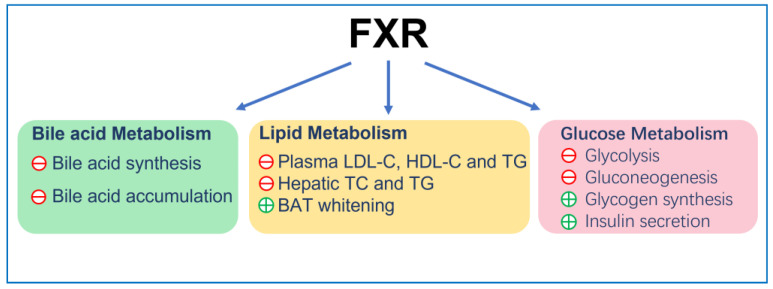
The general function of FXR. ㊉ indicates stimulation; ㊀ indicates inhibition.

FXR reduces the plasma low-density lipoprotein cholesterol (LDL-C) level [67] by inducing the internalization and degradation of the LDL particle [68]. Moreover, it was found that FXR knockout mice displayed elevated plasma high-density lipoprotein cholesterol (HDL-C) due to the reduced expression of reverse cholesterol transport gene, scavenger receptor class B member 1 (SCARB1) and ATP-binding cassette (ABC) transporters G5 (ABCG5) and G8 (ABCG8), which facilitated the removal of HDL-C from the blood [69,70]. Finally, FXR can also reduce the accumulation of cholesterol in hepatocytes and renal epithelial cells. This can be achieved by decreasing cholesterol synthesis via inhibition of the expression of sterol-regulatory element-binding protein 2 (SREBP-2) and β-Hydroxy β-methylglutaryl-CoA (HMG-CoA) [71] and promoting cholesterol efflux via increasing expression of ATP-binding cassette transporterA1 (ABCA1) [72]. On the other hand, FXR lowered hepatic and renal triglyceride accumulation and plasma triglyceride levels in insulin resistance models, such as ob/ob and KK-Ay mice [59], by reducing the expression of fatty acid synthase (FAS) and acetyl CoA carboxylase (ACC). This was achieved by inhibiting SREBP-1c and carbohydrate response element binding protein (ChREBP) [73,74,75], promoting triglycerides clearance by increasing fatty acids oxidation via PPARα/γ- carnitine palmitoyltransferase I (CPT1) axis [76,77] and reducing fatty acid uptake by reducing the expression of CD36 [78]. In adipocyte, FXR induces brown adipose tissue (BAT) whitening, presenting with large intracellular lipid droplets and extracellular collagen deposition as a result of activation of stearoyl-coenzyme A desaturase (SCD) expression via PPARγ activation [79,80,81,82] (Figure 1).

## 5. FXR and Renal Physiology

The kidney is responsible for the excretion of urine, allowing toxins, metabolic waste products and excess ion to be excreted while maintaining essential substances in the blood and regulating several body systems such as intra and extracellular volume status, acid-base status, calcium and phosphate metabolism or erythropoiesis. It also produces renin for blood-pressure regulation and expresses 1α hydroxylase for conversion of vitamin D to its active form. Each day, an adult human produces ∼1.5 L of urine despite 180 L of fluid being filtered through the glomerular basement membrane. Approximately 99% of glomerular filtrate is reabsorbed constitutively along successive segments of the nephron. In renal medullary collecting duct cells (MCDs), water transport is tightly regulated by arginine vasopressin (AVP), a circulating hormone also known as antidiuretic hormone (ADH). Dehydration causes a decrease in fluid volume and an increase in blood osmolality, and this water-restricted condition promotes the release of hypothalamic AVP into the blood. AVP stimulates the water reabsorption in kidney by increasing gene expression and apical membrane targeting of aquaporin 2 (AQP2) [83]. 

FXR is widely expressed in all segments of renal tubules, with relatively higher expression in the proximal tubules and thick ascending limbs, followed by the distal tubules, thin descending limbs and collecting ducts. FXR plays a critical role in the regulation of urine volume, and its activation increases urinary concentrating capacity, mainly by upregulating its target gene AQP2 expression in the collecting ducts [84]. Renal medulla is a unique tissue in which residing cells including MCDs are exposed to the harsh hypertonic and hypoxic environment and must survive significant increases in NaCl and urea concentrations during antidiuresis. FXR can ameliorate hypertonic apoptosis of MCDs by activating tonicity response enhancer-binding protein (TonEBP) and its target gene, crystallin zeta (CRYZ) [85,86]. It is reported that deletion of transcription factor hepatocyte nuclear factor-1β (HNF-1β) in the mouse renal collecting ducts (CDs) induced polyuria and polydipsia. Chromatin immunoprecipitation and sequencing experiments revealed HNF-1β binding to FXR gene promoter. This study revealed a novel transcriptional regulator of FXR in maintaining urine concentration [87]. These studies have shown that FXR plays a critical role in urine concentration by increasing water reabsorption and promoting the survival of MCDs in a dehydrated state. In addition, FXR may be a potential target for the treatment of diseases such as hepatorenal syndrome and cholestasis, which have high circulating bile acid levels. 

Several studies revealed that bile acid transporters were expressed in the kidney. Bile acids are reabsorbed by ASBT in the apical membrane of proximal tubular cells and excreted by MRP2 and MRP4 in the apical membrane and OSTα/OSTβ in the basolateral membrane, while MRP3 is not expressed to a significant level in mouse kidney [88]. The activation of renal FXR promotes bile acid excretion from urine by increasing the expression of MRP4 in the apical membranes [89] and OSTα/OSTβ in the basolateral membrane [90]. FXR have little effect on renal MRP2 and ASBT [88,90,91]. These observations suggested that in the kidney, FXR may contribute to urinary bile acid elimination, especially during cholestasis.

**Figure 2:**
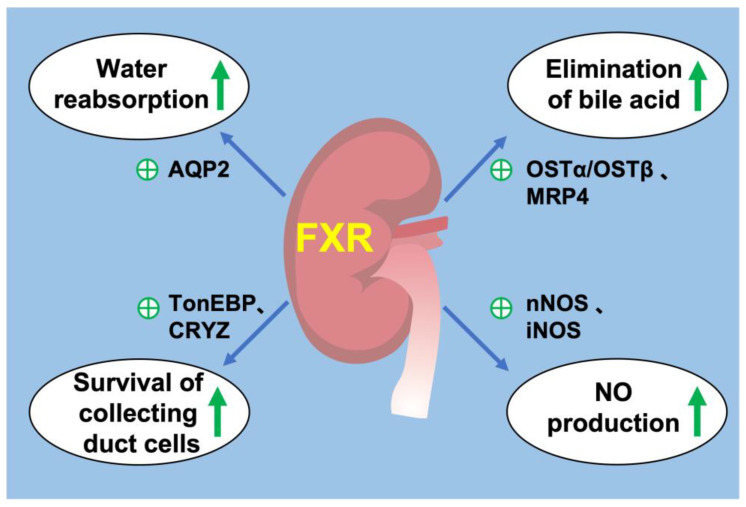
FXR and renal physiology. (1) FXR activation promotes water reabsorption in renal collecting duct by upregulating AQP2 expression. (2) FXR promotes the elimination of bile acids from the kidney by promoting MRP4 in the apical membrane and OSTα/OSTβ in the basolateral membrane. (3) FXR increases the expression of TonEBP and CRYZ to promote the survival of renal collecting duct under hyperosmotic stress. (4) FXR promotes the production of NO by increasing the expression of nNOS and iNOS in the kidney. ㊉ indicates stimulation. green arrow indicates upregulation.

The kidney plays a central role in blood-pressure regulation by causing vasoconstriction and controlling circulating blood volume. Zhu et al. reported that in the mouse renal collecting duct cells mIMCD-K2, FXR agonists promoted NO production by enhancing the expression of neuronal nitric oxide synthase (nNOS) and inducible nitric oxide synthase (iNOS), whereas this effect was diminished by FXR knockdown [92]. In addition, they also investigated the anti-hypertensive effect of renal FXR on a hypertension mouse model induced by 20% fructose in drinking water with 4% sodium chloride in diet (HFS) for 8 weeks. They found that renal FXR overexpression significantly attenuated hypertension and increased renal NO levels. Moreover, chenodeoxycholic acid (CDCA), a natural ligand of FXR, attenuated elevated blood pressure in spontaneously hypertensive rats (SHR), partially through stimulating endothelial nitric oxide synthase (eNOS) expression [93,94]. These findings support a potential role of FXR in regulation of blood pressure and development of treatment for hypertension (Figure 2).

## 6. FXR and Kidney Diseases

### 6.1. Acute Kidney Injury

Acute kidney injury is a syndrome characterized by rapid loss of renal excretory function, usually diagnosed by the accumulation of end products of nitrogen metabolism (urea and creatinine) or decreased urine output. Classification of AKI includes pre-renal, intrinsic and post-renal kidney injury. Current models of AKI can be induced by ischemia–reperfusion (pre-renal acute kidney injury), injection of drugs, toxins or endogenous toxins such as cisplatin and triptolide (intrinsic acute kidney injury) and ureteral obstruction (post-renal acute kidney injury) [95]. FXR plays an important role in various types of acute kidney injury.

In renal ischemia–reperfusion (I/R) injury mice models, OCA and GW4064 can improve renal structural and function damage, decrease inflammation and apoptosis and attenuate renal oxidative and ER stress. In primary cultured mouse renal proximal tubular cells, FXR activation resulted in markedly decreased oxidative stress, mitochondrial damage and ER stress in response to hypoxia [96]. OCA also prevented the subsequent progression of AKI to chronic kidney disease (CKD) by ameliorating glomerulosclerosis and interstitial fibrosis [96,97]. Moreover, alisol B 23-acetate (ABA), a major active triterpenoid extracted from alismatis rhizome, can activate FXR and improve renal function, reducing renal tubular apoptosis by ameliorating oxidative stress and suppressing inflammatory factor expression [98]. Kim et al. found that FXR deficiency mice exhibited increased renal apoptosis and autophagy compared to wildtype mice. Meanwhile, in HK2 cell (human renal proximal tubular epithelial cells), treatment with GW4064 and OCA inhibited hypoxia-induced autophagy [97]. Controversially, it is reported that FXR-deficient mice have less kidney dysfunction, with significantly lower levels of Cr and BUN compared with wildtype mice after I/R injury. FXR deficiency attenuated I/R (hypoxia for 24h followed by 6 h of reoxygenation)-induced apoptosis in HK2 cells by increasing PI3k/Akt-mediated Bad phosphorylation [99]. Based on these studies, the effects of FXR on I/R induced renal injury and cell apoptosis are inconclusive, and more evidence is needed. AKI induced by I/R involves multiple mechanisms, including renal tubular epithelial cells and immune/inflammatory cells. Cell-specific knockout would help elucidate the role of FXR in renal I/R injury. 

In cisplatin-induced AKI, knockout of the FXR gene aggravated renal injury, the mechanism of which might be related to inhibiting autophagy and promoting apoptosis [100]. Meanwhile, OCA protected against cisplatin-induced inflammation, apoptosis and fibrosis in the kidney by regulating small heterodimer partner (SHP) [101]. In addition to the important roles of apoptosis and autophagy in acute kidney injury, recent studies have shown that ferroptosis is also involved in its occurrence. In cisplatin-induced AKI, the expression of FXR and GPX4 (the central regulator of ferroptosis) decreased while lipid peroxidation increased. FXR agonist GW4064 protected against cisplatin-induced acute kidney injury by regulating the transcription of ferroptosis-related genes [102]. Current studies showed that FXR agonist GW4064 reduced renal lipid deposition by increasing fatty acid oxidation via PPARγ, thereby ameliorating cisplatin-induced acute kidney injury [77]. Other studies reported that OCA pretreatment alleviated LPS-induced renal dysfunction and pathological damage by repressing inflammation and oxidation [103]. Dioscin, a natural saponin derived from various herbs, acted as a novel and potent FXR agonist, which suppressed inflammation and oxidative stress against doxorubicin-induced nephrotoxicity [104]. Therefore, FXR offers a promising therapeutic target for drug-induced AKI.

### 6.2. Chronic Kidney Disease 

Chronic kidney disease is defined as kidney damage or glomerular filtration rate (GFR) < 60 mL/min/1.73 m^2^ for 3 months or more, irrespective of cause. A progressive disease with no cure and high morbidity and mortality, chronic kidney disease is common in the general adult population, especially in people with diabetes and hypertension [105]. 

Diabetic kidney disease is the leading cause of CKD worldwide [106]. It is characterized by glomerular hypertrophy, mesangial expansion, tubulo-interstitial fibrosis and inflammation, glomerulosclerosis, kidney fibrosis and podocyte loss [107]. FXR is markedly decreased in both glomeruli and tubules in human kidney with diabetic kidney disease [108]. In db/db mice with type 2 diabetes, FXR agonist GW4064 or INT-767 attenuated podocytes injury, glomerulosclerosis, mesangial expansion and tubulointerstitial fibrosis by downregulating the pro-fibrotic and pro-inflammatory genes. They also significantly attenuated abnormal lipid metabolism by reducing the gene expression related to synthesis of fatty acids such as SREBP-1, while increasing the expression of ABCA-1, a modulator for cholesterol efflux [74,109,110]. In addition, tauroursodeoxycholic acid (TUDCA) and WAY-362450 (FXR-450/XL335)—both potent agonists of FXR—alleviated glomerular and tubular damage by decreasing serum TG levels and inhibiting ER stress in db/db mice [111,112]. Moreover, in db/db mice, GW4064 ameliorated the progression of diabetic nephropathy by downregulating visfatin expression, which is a recently discovered adipocytokine that has been shown to have an important role in the pathogenesis of diabetic kidney disease [113]. OCA also improved renal injury by decreasing proteinuria, glomerulosclerosis and tubulointerstitial fibrosis in mice with type 1 diabetes [114]. FXR activation-mitigated tacrolimus induced diabetes mellitus by inhibiting gluconeogenesis and promoting glucose uptake of renal cortex proximal tubule epithelial cells in a PGC1α/FOXO1-dependent manner [115]. In summary, FXR agonists may prevent diabetic kidney disease by improving renal lipid accumulation, glucose metabolism disorders, inflammation and fibrosis.

Obesity-related renal disease is associated with structural and functional changes in various kinds of renal cells, including mesangial cells, podocytes and proximal tubular cells. Obesity has become a worldwide epidemic and is an independent risk factor for development and progression of CKD. Renal lipid metabolism disorder is the main feature of obesity-related renal disease. In mice with diet-induced obesity, INT-767 and OCA prevented abnormal renal lipid metabolism, mitochondrial dysfunction, oxidative stress, inflammation and fibrosis [74,116]. Trans chalcone is a flavonoids precursor that activated FXR, significantly inhibited HFD-induced decreased insulin sensitivity and increased expression levels of SREBP-1c, FAS, MAD-3 and NGAL, thereby acting as a renal protective agent [75]. In addition, the offspring of obese mothers are more likely to develop impaired glucose tolerance and CKD, with associated downregulation of renal FXR expression and upregulation of monocyte chemoattractant protein-1 (MCP-1) and transforming growth factor-β1 (TGF-β1) [117]. In fructose-fed Wistar rats, high-fructose feeding might cause lipid nephrotoxicity through downregulated FXR, but FXR agonist CDCA modulated renal lipid metabolism, decreased proteinuria, improves renal fibrosis, inflammation and oxidative stress [118]. In uninephrectomized obese mice, OCA attenuated renal injury, renal lipid accumulation, apoptosis and lipid peroxidation [119]. Thus, activation of FXR may be a novel strategy for treatment of obesity-associated renal disease.

Obstructive kidney disease is typically simulated via ureteral obstruction (UUO) in animal models, causing renal fibrosis, a common pathway for most chronic kidney disease to progress to end-stage renal failure. In UUO mice, FXR agonist protected against renal fibrosis and downregulated Smad3 expression, which is a critical signaling protein in renal fibrosis [120]. In addition, the activation of FXR suppressed renal fibrosis by inhibiting the phosphorylation of Tyr416-Src (proto-oncogene tyrosine-protein kinase) and increasing Ser127 phosphorylation and cytosolic accumulation of yes-associated protein (YAP) [121]. Another FXR agonist, EDP-305, also reduced interstitial renal fibrosis by increasing inhibitory YAP phosphorylation in UUO mice [122]. Therefore, targeting FXR protected against renal fibrosis in a ureteral obstruction animal model.

Hepatorenal syndrome (HRS) is the most significant hepatorenal disorder occurring in patients with advanced cirrhosis. In a rat model of HRS induced by bile duct ligation for 6 weeks, OCA treatment significantly normalized portal hypertension, glomerular filtration rate, urine output, renal blood flow, decreased ascites, renal vascular resistance, serum creatinine and the release of renal tubular damage markers, including urinary neutrophil gelatinase-associated lipocalin (uNGAL) and kidney injury moleculae-1 (uKim-1) through the inhibition of renal 8-iso-PGF2α production and the downregulation of the COX-TXA2 pathway [123]. NAFLD and NASH are also associated with an increased risk of chronic kidney disease (CKD), while Vonafexor, an FXR agonist being developed for patients with NASH, can not only improve liver fat deposition, but can also increase glomerular filtration rate, thereby improving renal function [124].

**Figure 3:**
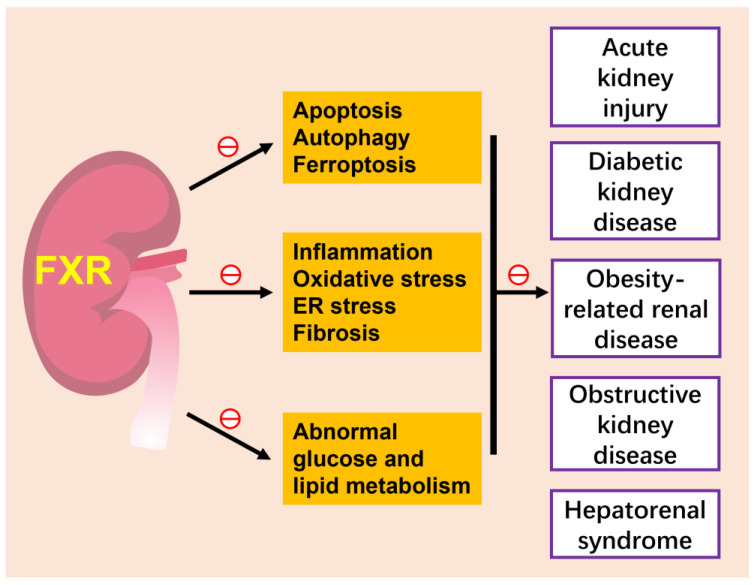
FXR and kidney diseases. The activation of FXR attenuates various kidney diseases, such as acute kidney injury, diabetic kidney disease, obesity-related kidney disease and hepatorenal syndrome, by inhibiting several cell death modes of renal tubular epithelium, including apoptotic, autophagy and ferroptosis, ameliorating inflammation, oxidative stress and fibrosis and improving abnormal glucose and lipid metabolism. The role of FXR in renal neoplasia is slightly unclear and is not included in this figure. ㊀ indicates inhibition.

Renal cell carcinoma (RCC) is the most common type of kidney cancer [125]. Some studies have found that FXR plays different roles in normal cells and tumor cells. In HK-2 cells, FXR stimulated cell differentiation by decreasing Oct3/4 level [126,127,128,129], which is a cell differentiation marker, but had no effect on the differentiation of renal adenocarcinoma cells. Controversially, FXR also stimulated the cell growth of renal adenocarcinoma cells by downregulating cyclin-dependent kinase (CDK) inhibitors p16/INK4a and p21/Cip1, but had no effect in the normal renal cell-derived cell line [130]. Recently, a cohort of adult clear cell renal cell carcinoma (ccRCC) patients revealed that FXR activation inhibits the progression of ccRCC [131]. The role of FXR in renal neoplasia needs further study (Figure 3).

## 7. Conclusions and Outlook

FXR plays crucial roles in regulating bile acid metabolism, cholesterol homeostasis, glucose and lipid metabolism. Emerging evidence demonstrates that FXR agonists are functional for metabolic syndrome and are considered potential therapeutic agents. FXR is widely expressed in the kidneys. Under physiological conditions, it plays important roles in urine concentration by promoting water reabsorption and cell survival under hyperosmotic stress. Under pathological conditions, FXR mitigates apoptosis, autophagy and ferroptosis of tubular epithelial cells, attenuates abnormal glucose and lipid metabolism and improves inflammation, oxidative stress, ER stress and fibrosis in various kidney diseases. Thus, targeting FXR has great application prospects in the treatment of various kidney diseases. However, little is known about the mechanisms by which the different FXR isoforms regulate specific genes and how the expression of these target genes affects the occurrence and development of diseases. Moreover, the kidney is a complicated organ made up of multiple cell types; the cell-specific functions of FXR also need to be elucidated in the future.

## Figures and Tables

**Table 1:** FXR isoforms.

Nuclear Receptors	Official Symbol	Isoforms	Exon Count	Insertion	Organism
FXRα	*NRIH4*	FXRα1 FXRα2 FXRα3 FXRα4	11 11 9 9	+ − + −	*Rodents* and *primates*
FXRβ	*NRIH5*	Five splice variants were isolated, not named	11	/	*Rodents*

Insertion: 12-bp amino acid sequence (MYTG), including (+), lacking (−). FXRα5-8 are four novel splice variants recently found in human hepatocytes and therefore not included in the table. FXRβ is a pseudogene in primates.

**Table 2:** FXR agonist clinical trials.

Agonists	Clinical Trial Status	NCT Identifiers	Indication	References
Obeticholic acid	FDA approved	NCT02308111	PBC, PSC, NASH	[21]
	Phase II	NCT01585025		
	Phase III	NCT02548351		
EDP-305	Phase I Phase II	NCT03748628	PBC, NASH	[22]
		NCT03394924		
MET409	Phase II	NCT04702490	NASH, T2DM	[27]
Cilofexor	Phase II Phase III	NCT02781584 NCT03890120	PBC, PSC, NASH	[24]
Tropifexor (LY2562175/LJN452)	Phase II	NCT02516605	PBC, NASH	[25]
Nidufexor	Phase II	NCT03804879	Diabetic Nephropathy	[26]
TERN-101(LMB-763)	Phase II	NCT04328077	NASH	[28]
EDP-297	Phase I	NCT04559126	NASH	[30]
PX-104	Phase II	NCT01999101	NAFLD	[32,33]
Vonafexor (EYP001)	Phase II Phase II	NCT03812029 NCT04365933	NASH Chronic Hepatitis B	[34]

PSC—Primary Sclerosing Cholangitis; NAFLD—non-alcoholic fatty liver disease.

**Table 3:** FXR antagonist clinical trials.

Antagonists	Clinical Trial Status	NCT Identifiers	Indication	References
Ursodeoxycholic acid	Phase IV Phase IV	NCT04617561 NCT04977661	NASH, PBC	[40,41]
Ivermectin	Phase III	NCT04529525	COVID-19	[42,43]
Benzamide	Phase III	NCT00534573	Clozapine-induced Hypersalivation	[44,45]

## Data Availability

Not applicable.

## References

[1] Schulman I.G. (2010). Nuclear receptors as drug targets for metabolic disease. Adv. Drug Deliv. Rev..

[2] Seol W., Choi H.S., Moore D.D. (1995). Isolation of proteins that interact specifically with the retinoid X receptor: Two novel orphan receptors. Mol. Endocrinol..

[3] Forman B.M., Goode E., Chen J., Oro A.E., Bradley D.J., Perlmann T., Noonan D.J., Burka L.T., McMorris T., Lamph W.W. (1995). Identification of a nuclear receptor that is activated by farnesol metabolites. Cell.

[4] Boesjes M., Bloks V.W., Hageman J., Bos T., van Dijk T.H., Havinga R., Wolters H., Jonker J.W., Kuipers F., Groen A.K. (2014). Hepatic Farnesoid X-Receptor Isoforms α2 and α4 Differentially Modulate Bile Salt and Lipoprotein Metabolism in Mice. PLoS ONE.

[5] Zhang Z.D., Cayting P., Weinstock G., Gerstein M. (2007). Analysis of Nuclear Receptor Pseudogenes in Vertebrates: How the Silent Tell Their Stories. Mol. Biol. Evol..

[6] Mustonen E.-K., Lee S.M., Nieß H., Schwab M., Pantsar T., Burk O. (2021). Identification and characterization of novel splice variants of human farnesoid X receptor. Arch. Biochem. Biophys..

[7] Otte K., Kranz H., Kober I., Thompson P., Hoefer M., Haubold B., Remmel B., Voss H., Kaiser C., Albers M. (2003). Identification of farnesoid X receptor beta as a novel mammalian nuclear receptor sensing lanosterol. Mol. Cell. Biol..

[8] Zhang Y., Kast-Woelbern H.R., Edwards P.A. (2003). Natural Structural Variants of the Nuclear Receptor Farnesoid X Receptor Affect Transcriptional Activation. J. Biol. Chem..

[9] Lien F., Berthier A., Bouchaert E., Gheeraert C., Alexandre J., Porez G., Prawitt J., Dehondt H., Ploton M., Colin S. (2014). Metformin interferes with bile acid homeostasis through AMPK-FXR crosstalk. J. Clin. Investig..

[10] Anbalagan M., Huderson B., Murphy L., Rowan B.G. (2012). Post-Translational Modifications of Nuclear Receptors and Human Disease. Nucl. Recept. Signal..

[11] Zhang Y., Castellani L.W., Sinal C.J., Gonzalez F.J., Edwards P.A. (2004). Peroxisome proliferator-activated receptor-gamma coactivator 1alpha (PGC-1alpha) regulates triglyceride metabolism by activation of the nuclear receptor FXR. Genes Dev..

[12] Gineste R., Sirvent A., Paumelle R., Helleboid S., Aquilina A., Darteil R., Hum D.W., Fruchart J.-C., Staels B. (2008). Phosphorylation of Farnesoid X Receptor by Protein Kinase C Promotes Its Transcriptional Activity. Mol. Endocrinol..

[13] Berrabah W., Aumercier P., Gheeraert C., Dehondt H., Bouchaert E., Alexandre J., Ploton M., Mazuy C., Caron S., Tailleux A. (2014). Glucose sensing O-GlcNAcylation pathway regulates the nuclear bile acid receptor farnesoid X receptor (FXR). Hepatology.

[14] Kemper J.K., Xiao Z., Ponugoti B., Miao J., Fang S., Kanamaluru D., Tsang S., Wu S.-Y., Chiang C.-M., Veenstra T.D. (2009). FXR Acetylation Is Normally Dynamically Regulated by p300 and SIRT1 but Constitutively Elevated in Metabolic Disease States. Cell Metab..

[15] Olefsky J.M. (2001). Nuclear Receptor Minireview Series. J. Biol. Chem..

[16] Chai S.C., Chen T., Kenakin T. (2022). 1.07—Nuclear Receptors. Comprehensive Pharmacology.

[17] Wurtz J.M., Bourguet W., Renaud J.P., Vivat V., Chambon P., Moras D., Gronemeyer H. (1996). A canonical structure for the ligand-binding domain of nuclear receptors. Nat. Struct. Biol..

[18] Weikum E.R., Liu X., Ortlund E.A. (2018). The nuclear receptor superfamily: A structural perspective. Protein Sci..

[19] Laffitte B.A., Kast H.R., Nguyen C.M., Zavacki A.M., Moore D.D., Edwards P.A. (2000). Identification of the DNA Binding Specificity and Potential Target Genes for the Farnesoid X-activated Receptor. J. Biol. Chem..

[20] Wang H., Chen J., Hollister K., Sowers L.C., Forman B.M. (1999). Endogenous Bile Acids Are Ligands for the Nuclear Receptor FXR/BAR. Mol. Cell.

[21] Younossi Z.M., Ratziu V., Loomba R., Rinella M., Anstee Q.M., Goodman Z., Bedossa P., Geier A., Beckebaum S., Newsome P.N. (2019). Obeticholic acid for the treatment of non-alcoholic steatohepatitis: Interim analysis from a multicentre, randomised, placebo-controlled phase 3 trial. Lancet.

[22] Ahmad A., Sanderson K., Dickerson D., Adda N. (2018). Pharmacokinetics, pharmacodynamics, and safety of EDP-305, in healthy and presumptive NAFLD subjects. J. Hepatol..

[23] Li W., Fu J., Cheng F., Zheng M., Zhang J., Liu G., Tang Y. (2012). Unbinding Pathways of GW4064 from Human Farnesoid X Receptor as Revealed by Molecular Dynamics Simulations. J. Chem. Inf. Model..

[24] Trauner M., Gulamhusein A., Hameed B., Caldwell S., Shiffman M.L., Landis C., Eksteen B., Agarwal K., Muir A., Rushbrook S. (2019). The Nonsteroidal Farnesoid X Receptor Agonist Cilofexor (GS-9674) Improves Markers of Cholestasis and Liver Injury in Patients with Primary Sclerosing Cholangitis. Hepatology.

[25] Badman M.K., Chen J., Desai S., Vaidya S., Neelakantham S., Zhang J., Gan L., Danis K., Laffitte B., Klickstein L.B. (2020). Safety, tolerability, pharmacokinetics, and pharmacodynamics of the novel non–bile acid FXR agonist tropifexor (LJN452) in healthy volunteers. Clin. Pharmacol. Drug Dev..

[26] Chianelli D., Rucker P.V., Roland J., Tully D.C., Nelson J., Liu X., Bursulaya B., Hernandez E.D., Wu J., Prashad M. (2020). Nidufexor (LMB763), a Novel FXR Modulator for the Treatment of Nonalcoholic Steatohepatitis. J. Med. Chem..

[27] Harrison S.A., Bashir M.R., Lee K.-J., Shim-Lopez J., Lee J., Wagner B., Smith N.D., Chen H.C., Lawitz E.J. (2021). A structurally optimized FXR agonist, MET409, reduced liver fat content over 12 weeks in patients with non-alcoholic steatohepatitis. J. Hepatol..

[28] Wang Y., Crittenden D.B., Eng C., Zhang Q., Guo P., Chung D., Fenaux M., Klucher K., Jones C., Jin F. (2021). Safety, Pharmacokinetics, Pharmacodynamics, and Formulation of Liver-Distributed Farnesoid X-Receptor Agonist TERN-101 in Healthy Volunteers. Clin. Pharmacol. Drug Dev..

[29] Zhang S., Wang J., Liu Q., Harnish D.C. (2009). Farnesoid X receptor agonist WAY-362450 attenuates liver inflammation and fibrosis in murine model of non-alcoholic steatohepatitis. J. Hepatol..

[30] Marotta C., Ahmad A., Luo E., Oosterhaven J., van Marle S., Adda N. (2022). EDP-297: A novel, farnesoid X receptor agonist-Results of a phase I study in healthy subjects. Clin. Transl. Sci..

[31] Al-Khaifi A., Rudling M., Angelin B. (2018). An FXR Agonist Reduces Bile Acid Synthesis Independently of Increases in FGF19 in Healthy Volunteers. Gastroenterology.

[32] Traussnigg S., Halilbasic E., Hofer H., Munda P., Stojakovic T., Fauler G., Kashofer K., Krssak M., Wolzt M., Trauner M. (2020). Open-label phase II study evaluating safety and efficacy of the non-steroidal farnesoid X receptor agonist PX-104 in non-alcoholic fatty liver disease. Wien. Klin. Wochenschr..

[33] Schwabl P., Hambruch E., Seeland B.A., Hayden H., Wagner M., Garnys L., Strobel B., Schubert T.-L., Riedl F., Mitteregger D. (2017). The FXR agonist PX20606 ameliorates portal hypertension by targeting vascular remodelling and sinusoidal dysfunction. J. Hepatol..

[34] Erken R., Andre P., Roy E., Kootstra N., Barzic N., Girma H., Laveille C., Radreau-Pierini P., Darteil R., Vonderscher J. (2021). Farnesoid X receptor agonist for the treatment of chronic hepatitis B: A safety study. J. Viral Hepat..

[35] Gege C., Hambruch E., Hambruch N., Kinzel O., Kremoser C. (2019). Nonsteroidal FXR Ligands: Current Status and Clinical Applications. Handb. Exp. Pharm..

[36] Li F., Jiang C., Krausz K.W., Li Y., Albert I., Hao H., Fabre K.M., Mitchell J.B., Patterson A.D., Gonzalez F.J. (2013). Microbiome remodelling leads to inhibition of intestinal farnesoid X receptor signalling and decreased obesity. Nat. Commun..

[37] Jiang C., Xie C., Lv Y., Li J., Krausz K.W., Shi J., Brocker C.N., Desai D., Amin S.G., Bisson W.H. (2015). Intestine-selective farnesoid X receptor inhibition improves obesity-related metabolic dysfunction. Nat. Commun..

[38] Mueller M., Thorell A., Claudel T., Jha P., Koefeler H., Lackner C., Hoesel B., Fauler G., Stojakovic T., Einarsson C. (2015). Ursodeoxycholic acid exerts farnesoid X receptor-antagonistic effects on bile acid and lipid metabolism in morbid obesity. J. Hepatol..

[39] Brevini T., Maes M., Webb G.J., John B.V., Fuchs C.D., Buescher G., Wang L., Griffiths C., Brown M.L., Scott W.E. (2022). FXR inhibition may protect from SARS-CoV-2 infection by reducing ACE2. Nature.

[40] Nakano L.A., Cançado E.L.R., Chaves C.E., Madeira M.C.V., Katayose J.T., Nabeshima M.A., Fossaluza V., Uhrigshardt G.G., Liting Z., Pinto V.B. (2020). A randomized crossover trial to assess therapeutic efficacy and cost reduction of acid ursodeoxycholic manufactured by the university hospital for the treatment of primary biliary cholangitis. BMC Gastroenterol..

[41] Younossi Z., Tacke F., Arrese M., Sharma B.C., Mostafa I., Bugianesi E., Wong V.W.-S., Yilmaz Y., George J., Fan J. (2019). Global Perspectives on Nonalcoholic Fatty Liver Disease and Nonalcoholic Steatohepatitis. Hepatology.

[42] Hill A., Mirchandani M. (2022). The dangers of non-randomized, observational studies: Experience from the COVID-19 epidemic. J. Antimicrob. Chemother..

[43] Naggie S., Boulware D.R., Lindsell C.J., Stewart T.G., Lim S.C., Cohen J., Kavtaradze D., Amon A.P., Gabriel A., Gentile N. (2022). Effect of Ivermectin 600 μg/kg for 6 days vs. Placebo on Time to Sustained Recovery in Outpatients with Mild to Moderate COVID-19: A Randomized Clinical Trial. medRxiv.

[44] Song K., Xu X., Liu P., Chen L., Shen X., Liu J., Hu L. (2015). Discovery and SAR study of 3-(tert-butyl)-4-hydroxyphenyl benzoate and benzamide derivatives as novel farnesoid X receptor (FXR) antagonists. Bioorganic Med. Chem..

[45] Kreinin A., Miodownik C., Sokolik S., Shestakova D., Libov I., Bergman J., Lerner V. (2010). Amisulpride versus moclobemide in treatment of clozapine-induced hypersalivation. World J. Biol. Psychiatry.

[46] Sinal C.J., Tohkin M., Miyata M., Ward J.M., Lambert G., Gonzalez F.J. (2000). Targeted Disruption of the Nuclear Receptor FXR/BAR Impairs Bile Acid and Lipid Homeostasis. Cell.

[47] Lu T.T., Makishima M., Repa J., Schoonjans K., Kerr T.A., Auwerx J., Mangelsdorf D.J. (2000). Molecular Basis for Feedback Regulation of Bile Acid Synthesis by Nuclear Receptors. Mol. Cell.

[48] Goodwin B., Jones S.A., Price R.R., Watson M.A., McKee D.D., Moore L.B., Galardi C., Wilson J.G., Lewis M.C., Roth M.E. (2000). A Regulatory Cascade of the Nuclear Receptors FXR, SHP-1, and LRH-1 Represses Bile Acid Biosynthesis. Mol. Cell.

[49] Inagaki T., Choi M., Moschetta A., Peng L., Cummins C.L., McDonald J.G., Luo G., Jones S.A., Goodwin B., Richardson J.A. (2005). Fibroblast growth factor 15 functions as an enterohepatic signal to regulate bile acid homeostasis. Cell Metab..

[50] Denson L.A., Sturm E., Echevarria W., Zimmerman T.L., Makishima M., Mangelsdorf D.J., Karpen S.J. (2001). The Orphan Nuclear Receptor, shp, Mediates Bile Acid-Induced Inhibition of the Rat Bile Acid Transporter, ntcp. Gastroenterology.

[51] Kast H.R., Goodwin B., Tarr P.T., Jones S.A., Anisfeld A.M., Stoltz C.M., Tontonoz P., Kliewer S., Willson T.M., Edwards P.A. (2002). Regulation of Multidrug Resistance-associated Protein 2 (ABCC2) by the Nuclear Receptors Pregnane X Receptor, Farnesoid X-activated Receptor, and Constitutive Androstane Receptor. J. Biol. Chem..

[52] Ananthanarayanan M., Balasubramanian N., Makishima M., Mangelsdorf D.J., Suchy F.J. (2001). Human Bile Salt Export Pump Promoter Is Transactivated by the Farnesoid X Receptor/Bile Acid Receptor. J. Biol. Chem..

[53] Liu Y., Binz J., Numerick M.J., Dennis S., Luo G., Desai B., MacKenzie K.I., Mansfield T.A., Kliewer S.A., Goodwin B. (2003). Hepatoprotection by the farnesoid X receptor agonist GW4064 in rat models of intra- and extrahepatic cholestasis. J. Clin. Investig..

[54] Boyer J.L., Trauner M., Mennone A., Soroka C.J., Cai S.-Y., Moustafa T., Zollner G., Lee J.Y., Ballatori N. (2006). Upregulation of a basolateral FXR-dependent bile acid efflux transporter OSTα-OSTβ in cholestasis in humans and rodents. Am. J. Physiol. Liver Physiol..

[55] Chen F., Ma L., Dawson P.A., Sinal C.J., Sehayek E., Gonzalez F.J., Breslow J., Ananthanarayanan M., Shneider B.L. (2003). Liver Receptor Homologue-1 Mediates Species- and Cell Line-specific Bile Acid-dependent Negative Feedback Regulation of the Apical Sodium-dependent Bile Acid Transporter. J. Biol. Chem..

[56] Grober J., Zaghini I., Fujii H., Jones S.A., Kliewer S.A., Willson T.M., Ono T., Besnard P. (1999). Identification of a bile acid-responsive element in the human ileal bile acid-binding protein gene Involvement of the farnesoid X receptor/9-cis-retinoic acid receptor heterodimer. J. Biol. Chem..

[57] Landrier J.-F., Eloranta J.J., Vavricka S.R., Kullak-Ublick G.A. (2006). The nuclear receptor for bile acids, FXR, transactivates human organic solute transporter-α and-β genes. Am. J. Physiol. -Gastrointest. Liver Physiol..

[58] Dong R., Yang X., Wang C., Liu K., Liu Z., Ma X., Sun H., Huo X., Fu T., Meng Q. (2018). Yangonin protects against non-alcoholic fatty liver disease through farnesoid X receptor. Phytomedicine.

[59] Zhang Y., Lee F.Y., Barrera G., Lee H., Vales C., Gonzalez F.J., Willson T.M., Edwards P.A. (2006). Activation of the nuclear receptor FXR improves hyperglycemia and hyperlipidemia in diabetic mice. Proc. Natl. Acad. Sci..

[60] Caron S., Samanez C.H., Dehondt H., Ploton M., Briand O., Lien F., Dorchies E., Dumont J., Postic C., Cariou B. (2013). Farnesoid X Receptor Inhibits the Transcriptional Activity of Carbohydrate Response Element Binding Protein in Human Hepatocytes. Mol. Cell. Biol..

[61] Ma K., Saha P.K., Chan L., Moore D.D. (2006). Farnesoid X receptor is essential for normal glucose homeostasis. J. Clin. Investig..

[62] Zhao T., Wang J., He A., Wang S., Chen Y., Lu J., Lv J., Li S., Wang J., Qian M. (2021). Mebhydrolin ameliorates glucose homeostasis in type 2 diabetic mice by functioning as a selective FXR antagonist. Metabolism.

[63] Seyer P., Vallois D., Poitry-Yamate C., Schütz F., Metref S., Tarussio D., Maechler P., Staels B., Lanz B., Grueter R. (2013). Hepatic glucose sensing is required to preserve β cell glucose competence. J. Clin. Investig..

[64] Renga B., Mencarelli A., Vavassori P., Brancaleone V., Fiorucci S. (2010). The bile acid sensor FXR regulates insulin transcription and secretion. Biochim. et Biophys. Acta (BBA)—Mol. Basis Dis..

[65] Kong X., Li B., Deng Y., Ma X. (2019). FXR Mediates Adenylyl Cyclase 8 Expression in Pancreatic *β*-Cells. J. Diabetes Res..

[66] Kong X., Tu Y., Li B., Zhang L., Feng L., Wang L., Zhang L., Zhou H., Hua X., Ma X. (2019). Roux-en-Y gastric bypass enhances insulin secretion in type 2 diabetes via FXR-mediated TRPA1 expression. Mol. Metab..

[67] Nakahara M., Fujii H., Maloney P.R., Shimizu M., Sato R. (2002). Bile Acids Enhance Low Density Lipoprotein Receptor Gene Expression via a MAPK Cascade-mediated Stabilization of mRNA. J. Biol. Chem..

[68] Langhi C., Le May C., Kourimate S., Caron S., Staels B., Krempf M., Costet P., Cariou B. (2008). Activation of the farnesoid X receptor represses PCSK9 expression in human hepatocytes. FEBS Lett..

[69] Lambert G., Amar M.J.A., Guo G., Brewer H.B., Gonzalez F.J., Sinal C.J. (2003). The Farnesoid X-receptor Is an Essential Regulator of Cholesterol Homeostasis. J. Biol. Chem..

[70] Byun S., Jung H., Chen J., Kim Y.-C., Kim D.-H., Kong B., Guo G., Kemper B., Kemper J.K. (2019). Phosphorylation of hepatic farnesoid X receptor by FGF19 signaling–activated Src maintains cholesterol levels and protects from atherosclerosis. J. Biol. Chem..

[71] Lai C.-R., Tsai Y.-L., Tsai W.-C., Chen T.-M., Chang H.-H., Changchien C.-Y., Wu S.-T., Wang H.-H., Chen Y., Lin Y.-H. (2022). Farnesoid X Receptor Overexpression Decreases the Migration, Invasion and Angiogenesis of Human Bladder Cancers via AMPK Activation and Cholesterol Biosynthesis Inhibition. Cancers.

[72] Singh A.B., Dong B., Kraemer F.B., Liu J. (2020). FXR activation promotes intestinal cholesterol excretion and attenuates hyperlipidemia in SR-B1-deficient mice fed a high-fat and high-cholesterol diet. Physiol. Rep..

[73] Watanabe M., Houten S.M., Wang L., Moschetta A., Mangelsdorf D.J., Heyman R.A., Moore D.D., Auwerx J. (2004). Bile acids lower triglyceride levels via a pathway involving FXR, SHP, and SREBP-1c. J. Clin. Investig..

[74] Wang X.X., Wang D., Luo Y., Myakala K., Dobrinskikh E., Rosenberg A.Z., Levi J., Kopp J.B., Field A., Hill A. (2017). FXR/TGR5 Dual Agonist Prevents Progression of Nephropathy in Diabetes and Obesity. J. Am. Soc. Nephrol..

[75] Alipour M.R., Jeddi S., Karimi-Sales E. (2020). Trans-Chalcone inhibits high-fat diet-induced disturbances in FXR/SREBP-1c/FAS and FXR/Smad-3 pathways in the kidney of rats. J. Food Biochem..

[76] Liu Y., Song A., Yang X., Zhen Y., Chen W., Yang L., Wang C., Ma H. (2018). Farnesoid X receptor agonist decreases lipid accumulation by promoting hepatic fatty acid oxidation in db/db mice. Int. J. Mol. Med..

[77] Xu S., Jia P., Fang Y., Jin J., Sun Z., Zhou W., Li J., Zhang Y., Wang X., Ren T. (2022). Nuclear farnesoid X receptor attenuates acute kidney injury through fatty acid oxidation. Kidney Int..

[78] Ma Y., Huang Y., Yan L., Gao M., Liu D. (2013). Synthetic FXR Agonist GW4064 Prevents Diet-Induced Hepatic Steatosis and Insulin Resistance. Pharm. Res..

[79] Zhou W., Bandara S.R., Leal C., Anakk S. (2021). Lipid droplet structural remodeling in adipose tissue upon caloric excess. bioRxiv.

[80] Shinohara S., Fujimori K. (2020). Promotion of lipogenesis by PPARγ-activated FXR expression in adipocytes. Biochem. Biophys. Res. Commun..

[81] Van Zutphen T., Stroeve J.H.M., Yang J., Bloks V.W., Jurdzinski A., Roelofsen H., Huijkman N.C.A., van Dijk T.H., Vonk R.J., van Deursen J. (2019). FXR overexpression alters adipose tissue architecture in mice and limits its storage capacity leading to metabolic derangements [S]. J. Lipid Res..

[82] Yang J., de Vries H., Mayeuf-Louchart A., Stroeve J., Bloks V., Koehorst M., Duez H., Staels B., Kuipers F., van Zutphen T. (2023). Role of bile acid receptor FXR in development and function of brown adipose tissue. Biochim. et Biophys. Acta (BBA)—Mol. Cell Biol. Lipids.

[83] Petrillo F., Chernyakov D., Esteva-Font C., Poulsen S.B., Edemir B., Fenton R.A. (2022). Genetic deletion of the nuclear factor of activated T cells 5 in collecting duct principal cells causes nephrogenic diabetes insipidus. FASEB J..

[84] Zhang X., Huang S., Gao M., Liu J., Jia X., Han Q., Zheng S., Miao Y., Li S., Weng H. (2014). Farnesoid X receptor (FXR) gene deficiency impairs urine concentration in mice. Proc. Natl. Acad. Sci. USA.

[85] Alam G., Luan Z., Gul A., Lu H., Zhou Y., Huo X., Li Y., Du C., Luo Z., Zhang H. (2020). Activation of farnesoid X receptor (FXR) induces crystallin zeta expression in mouse medullary collecting duct cells.. Pflügers Arch. Eur. J. Physiol..

[86] Xu S., Huang S., Luan Z., Chen T., Wei Y., Xing M., Li Y., Du C., Wang B., Zheng F. (2018). Farnesoid X receptor is essential for the survival of renal medullary collecting duct cells under hypertonic stress. Proc. Natl. Acad. Sci. USA.

[87] Aboudehen K., Noureddine L., Cobo-Stark P., Avdulov S., Farahani S., Gearhart M.D., Bichet D.G., Pontoglio M., Patel V., Igarashi P. (2017). Hepatocyte Nuclear Factor–1β Regulates Urinary Concentration and Response to Hypertonicity. J. Am. Soc. Nephrol..

[88] Zollner G., Fickert P., Fuchsbichler A., Silbert D., Wagner M., Arbeiter S., Gonzalez F.J., Marschall H.-U., Zatloukal K., Denk H. (2003). Role of nuclear bile acid receptor, FXR, in adaptive ABC transporter regulation by cholic and ursodeoxycholic acid in mouse liver, kidney and intestine. J. Hepatol..

[89] Marschall H.U., Wagner M., Bodin K., Zollner G., Fickert P., Gumhold J., Silbert D., Fuchsbichler A., Sjövall J., Trauner M. (2006). Fxr(-/-) mice adapt to biliary obstruction by enhanced phase I detoxification and renal elimination of bile acids. J. Lipid Res..

[90] Zollner G., Wagner M., Moustafa T., Fickert P., Silbert D., Gumhold J., Fuchsbichler A., Halilbasic E., Denk H., Marschall H.U. (2006). Coordinated induction of bile acid detoxification and alternative elimination in mice: Role of FXR-regulated organic solute transporter-alpha/beta in the adaptive response to bile acids. Am. J. Physiol. Gastrointest. Liver Physiol..

[91] Maeda T., Miyata M., Yotsumoto T., Kobayashi D., Nozawa T., Toyama K., Gonzalez F.J., Yamazoe Y., Tamai I. (2004). Regulation of Drug Transporters by the Farnesoid X Receptor in Mice. Mol. Pharm..

[92] Zhu Y., Tao Y., Wu C., Zeng Y., Du H., Xiang Q., Chen Y., Zhu Q., Wang L. (2022). Renal Farnesoid X Receptor improves high fructose-induced salt-sensitive hypertension in mice by inhibiting DNM3 to promote nitro oxide production. J. Hypertens..

[93] Li C., Li J., Weng X., Lan X., Chi X. (2015). Farnesoid X receptor agonist CDCA reduces blood pressure and regulates vascular tone in spontaneously hypertensive rats. J. Am. Soc. Hypertens..

[94] Ghebremariam Y.T., Yamada K., Lee J.C., Johnson C.L.C., Atzler D., Anderssohn M., Agrawal R., Higgins J.P., Patterson A.J., Böger R.H. (2013). FXR Agonist INT-747 Upregulates DDAH Expression and Enhances Insulin Sensitivity in High-Salt Fed Dahl Rats. PLoS ONE.

[95] Li M., Hu T., Tie C., Qu L., Zheng H., Zhang J. (2017). Quantitative Proteomics and Targeted Fatty Acids Analysis Reveal the Damage of Triptolide in Liver and Kidney. Proteomics.

[96] Gai Z., Chu L., Xu Z., Song X., Sun D., Kullak-Ublick G.A. (2017). Farnesoid X receptor activation protects the kidney from ischemia-reperfusion damage. Sci. Rep..

[97] Kim D.-H., Park J.S., Choi H.-I., Kim C.S., Bae E.H., Ma S.K., Kim S.W. (2021). The critical role of FXR is associated with the regulation of autophagy and apoptosis in the progression of AKI to CKD. Cell Death Dis..

[98] Luan Z.L., Ming W.H., Sun X.W., Zhang C., Zhou Y., Zheng F., Yang Y.L., Guan Y.F., Zhang X.Y. (2021). A naturally occurring FXR agonist, alisol B 23-acetate, protects against renal ischemia-reperfusion injury. Am. J. Physiol. Ren. Physiol..

[99] Xu Y., Li D., Wu J., Zhang M., Shao X., Xu L., Tang L., Zhu M., Ni Z., Zhang M. (2021). Farnesoid X receptor promotes renal ischaemia-reperfusion injury by inducing tubular epithelial cell apoptosis. Cell Prolif..

[100] Zhang L., Li A., Huang Z., Wang Y., Yi B. (2022). Knockout of farnesoid X receptor gene aggravates cisplatin-induced kidney injury. Zhong Nan Da Xue Xue Bao Yi Xue Ban.

[101] Bae E.H., Choi H.S., Joo S.Y., Kim I.J., Kim C.S., Choi J.S., Ma S.K., Lee J., Kim S.W. (2014). Farnesoid X Receptor Ligand Prevents Cisplatin-Induced Kidney Injury by Enhancing Small Heterodimer Partner. PLoS ONE.

[102] Kim D.-H., Choi H.-I., Park J.S., Kim C.S., Bae E.H., Ma S.K., Kim S.W. (2022). Farnesoid X receptor protects against cisplatin-induced acute kidney injury by regulating the transcription of ferroptosis-related genes. Redox Biol..

[103] Zhu J.-B., Xu S., Li J., Song J., Luo B., Song Y.-P., Zhang Z.-H., Chen Y.-H., Xie D.-D., Yu D.-X. (2018). Farnesoid X receptor agonist obeticholic acid inhibits renal inflammation and oxidative stress during lipopolysaccharide-induced acute kidney injury. Eur. J. Pharmacol..

[104] Zhang Y., Xu Y., Qi Y., Xu L., Song S., Yin L., Tao X., Zhen Y., Han X., Ma X. (2017). Protective effects of dioscin against doxorubicin-induced nephrotoxicity via adjusting FXR-mediated oxidative stress and inflammation. Toxicology.

[105] Kalantar-Zadeh K., Jafar T.H., Nitsch D., Neuen B.L., Perkovic V. (2021). Chronic kidney disease. Lancet.

[106] Collins A.J., Kasiske B., Herzog C., Chavers B., Foley R., Gilbertson D., Grimm R., Liu J., Louis T., Manning W. (2005). Excerpts from the United States Renal Data System 2004 Annual Data Report: Atlas of end-stage renal disease in the United States. Am. J. Kidney Dis..

[107] Alicic R.Z., Rooney M.T., Tuttle K.R. (2017). Diabetic Kidney Disease. Chall. Prog. Possibilities.

[108] Wang X.X., Edelstein M.H., Gafter U., Qiu L., Luo Y., Dobrinskikh E., Lucia S., Adorini L., D’Agati V.D., Levi J. (2015). G Protein-Coupled Bile Acid Receptor TGR5 Activation Inhibits Kidney Disease in Obesity and Diabetes. J. Am. Soc. Nephrol..

[109] Han S.Y., Song H.K., Cha J.J., Han J.Y., Kang Y.S., Cha D.R. (2021). Farnesoid X receptor (FXR) agonist ameliorates systemic insulin resistance, dysregulation of lipid metabolism, and alterations of various organs in a type 2 diabetic kidney animal model. Acta Diabetol..

[110] Jiang T., Wang X.X., Scherzer P., Wilson P., Tallman J., Takahashi H., Li J., Iwahashi M., Sutherland E., Arend L. (2007). Farnesoid X Receptor Modulates Renal Lipid Metabolism, Fibrosis, and Diabetic Nephropathy. Diabetes.

[111] Marquardt A., Al-Dabet M.M., Ghosh S., Kohli S., Manoharan J., ElWakiel A., Gadi I., Bock F., Nazir S., Wang H. (2017). Farnesoid X Receptor Agonism Protects against Diabetic Tubulopathy: Potential Add-On Therapy for Diabetic Nephropathy. J. Am. Soc. Nephrol..

[112] Evans M., Mahaney P., Marcucci L., Lai K., Wang S., Krueger J., Gardell S., Huard C., Martinez R., Vlasuk G. (2009). A synthetic farnesoid X receptor (FXR) agonist promotes cholesterol lowering in models of dyslipidemia. Am. J. Phys-iol.-Gastrointest. Liver Physiol..

[113] Zhou B., Feng B., Qin Z., Zhao Y., Chen Y., Shi Z., Gong Y., Zhang J., Yuan F., Mu J. (2016). Activation of farnesoid X receptor downregulates visfatin and attenuates diabetic nephropathy. Mol. Cell. Endocrinol..

[114] Wang X.X., Jiang T., Shen Y., Caldas Y., Miyazaki-Anzai S., Santamaria H., Urbanek C., Solis N., Scherzer P., Lewis L. (2010). Diabetic Nephropathy Is Accelerated by Farnesoid X Receptor Deficiency and Inhibited by Farnesoid X Receptor Activation in a Type 1 Diabetes Model. Diabetes.

[115] Li L., Zhao H., Chen B., Fan Z., Li N., Yue J., Ye Q. (2019). FXR activation alleviates tacrolimus-induced post-transplant diabetes mellitus by regulating renal gluconeogenesis and glucose uptake. J. Transl. Med..

[116] Wang X.X., Jiang T., Shen Y., Adorini L., Pruzanski M., Gonzalez F.J., Scherzer P., Lewis L., Miyazaki-Anzai S., Levi M. (2009). The farnesoid X receptor modulates renal lipid metabolism and diet-induced renal inflammation, fibrosis, and proteinuria. Am. J. Physiol. Physiol..

[117] Glastras S.J., Wong M.G., Chen H., Zhang J., Zaky A., Pollock C.A., Saad S. (2015). FXR expression is associated with dysregulated glucose and lipid levels in the offspring kidney induced by maternal obesity. Nutr. Metab..

[118] Hu Z., Ren L., Wang C., Liu B., Song G. (2012). Effect of Chenodeoxycholic Acid on Fibrosis, Inflammation and Oxidative Stress in Kidney in High-Fructose-Fed Wistar Rats. Kidney Blood Press. Res..

[119] Gai Z., Gui T., Hiller C., Kullak-Ublick G.A. (2016). Farnesoid X receptor protects against kidney injury in uninephrectomized obese mice. J. Biol. Chem..

[120] Zhao K., He J., Zhang Y., Xu Z., Xiong H., Gong R., Li S., Chen S., He F. (2016). Activation of FXR protects against renal fibrosis via suppressing Smad3 expression. Sci. Rep..

[121] Kim D.-H., Choi H.-I., Park J.S., Kim C.S., Bae E.H., Ma S.K., Kim S.W. (2019). Src-mediated crosstalk between FXR and YAP protects against renal fibrosis. FASEB J..

[122] Li S., Ghoshal S., Sojoodi M., Arora G., Masia R., Erstad D.J., Ferriera D.S., Li Y., Wang G., Lanuti M. (2019). The farnesoid X receptor agonist EDP-305 reduces interstitial renal fibrosis in a mouse model of unilateral ureteral obstruction. FASEB J..

[123] Tsai Y.-L., Liu C.-W., Hsu C.-F., Huang C.-C., Lin M.-W., Huang S.-F., Li T.-H., Lee K.-C., Hsieh Y.-C., Yang Y.-Y. (2020). Obeticholic acid ameliorates hepatorenal syndrome in ascitic cirrhotic rats by down-regulating the renal 8-iso-PGF2α-activated COX-TXA2 pathway. Clin. Sci..

[124] Ratziu V., Harrison S.A., Loustaud-Ratti V., Bureau C., Lawitz E., Abdelmalek M., Alkhouri N., Francque S., Girma H., Darteil R. (2022). Hepatic and renal improvements with FXR agonist vonafexor in individuals with suspected fibrotic NASH. J. Hepatol..

[125] Girisa S., Shabnam B., Monisha J., Fan L., Halim C.E., Arfuso F., Ahn K.S., Sethi G., Kunnumakkara A.B. (2019). Potential of Zerumbone as an Anti-Cancer Agent. Molecules.

[126] Fujino T., Sugizaki K., Kato R., Beppu M., Murakami S., Lee H., Oshima T., Hayakawa M. (2020). Farnesoid X receptor and liver X receptors regulate Oct3/4 expression by multiple feedback regulating system in normal renal-derived cells and renal adenocarcinoma cells. J. Toxicol. Sci..

[127] Niwa H., Miyazaki J., Smith A.G. (2000). Quantitative expression of Oct-3/4 defines differentiation, dedifferentiation or self-renewal of ES cells. Nat. Genet..

[128] Kanai D., Ueda A., Akagi T., Yokota T., Koide H. (2015). Oct3/4 directly regulates expression of E2F3a in mouse embryonic stem cells. Biochem. Biophys. Res. Commun..

[129] Tomizawa M., Shinozaki F., Motoyoshi Y., Sugiyama T., Yamamoto S., Ishige N. (2018). Oct3/4 is potentially useful for the suppression of the proliferation and motility of hepatocellular carcinoma cells. Oncol. Lett..

[130] Fujino T., Sakamaki R., Ito H., Furusato Y., Sakamoto N., Oshima T., Hayakawa M. (2017). Farnesoid X receptor regulates the growth of renal adenocarcinoma cells without affecting that of a normal renal cell-derived cell line. J. Toxicol. Sci..

[131] Strauss P., Rivedal M., Scherer A., Eikrem Ø., Nakken S., Beisland C., Bostad L., Flatberg A., Skandalou E., Beisvåg V. (2022). A multiomics disease progression signature of low-risk ccRCC. Sci. Rep..

